# Extended passaging of the SKOV3 ovarian cancer cell line leads to two phenotypically different strains

**DOI:** 10.1242/dmm.052451

**Published:** 2025-08-26

**Authors:** Eglė Žymantaitė, Migle Gabrielaite, Vita Pašukonienė, Agata Mlynska

**Affiliations:** ^1^ Laboratory of Immunology, National Cancer Institute, P. Baublio g. 3B, LT-08406 Vilnius, Lithuania; ^2^ Institute of Biosciences, Life Sciences Center, Vilnius University, Sauletekio 7, LT-10257 Vilnius, Lithuania; ^3^ Institute of Biotechnology, Life Sciences Center, Vilnius University, Sauletekio 7, LT-10257 Vilnius, Lithuania; ^4^ Department of Chemistry and Bioengineering, Vilnius Gediminas Technical University, Sauletekio al. 11, LT-10223 Vilnius, Lithuania

**Keywords:** Cancer cell lines, Ovarian cancer, Clonal evolution, Late passaging, SKOV3

## Abstract

Continuous passaging of cancer cell lines can drive phenotypic and genotypic divergence, potentially compromising the reliability of such models. In this study, we show that two late-passage strains (S1 and S2) of ovarian cancer cell line SKOV3, although authenticated via short tandem repeat (STR) profiling as identical, exhibit substantial differences in morphology, transcriptomic signatures, ability to form 3D cultures and chemotherapeutic responses. Notably, S1 formed compact 3D spheroids and exhibited enhanced epithelial-mesenchymal transition (EMT) pathway activity, whereas S2 displayed a more proliferative, MYC-driven phenotype with larger spheroid structures requiring higher seeding densities. Transcriptomic analysis revealed pathways associated with hypoxia, EMT and angiogenesis in 3D culture, highlighting the complexity introduced by dimensionality in tumour modelling. Critically, S1 showed higher sensitivity to doxorubicin than S2 (IC50 of 0.12 µM versus 1.28 µM, *P*=0.0001), indicating how clonal evolution can confound drug-response assays. Ultimately, our findings suggest that although STR profiling remains essential for cell line authentication, functionally distinct subpopulations can arise and coexist within the same culture, and their isolation may reveal divergent phenotypes that compromise reproducibility in preclinical cancer research.

## Teaser

**Summary:** Extended passaging of the SKOV3 ovarian cancer line can give rise to distinct strains that exhibit divergent morphologies, drug responses, transcriptomic profiles and 3D culture formation capabilities.

## INTRODUCTION

Cancer cell lines are one of the most important and commonly used models in oncology research, offering insights into tumour biology, therapeutic response and mechanisms of drug resistance. Their reliability and proper characterization are important in translating laboratory findings into effective treatments and for accurately modelling cancer behaviour *in vivo* (van Staveren et al., 2009). However, cancer cell lines are not stable; continuous passaging has been shown to induce phenotypic and genotypic changes due to genetic drift, epigenetic modifications and altered growth conditions. As demonstrated by Uri Ben-David and colleagues, such changes can have a significant impact on cellular morphology and drug response (Ben-David et al., 2018), thereby threatening the reproducibility and validity of research findings.

Accurate authentication of cancer cell lines is, therefore, a critical first step to ensure the reliability of experimental results. Misidentified or contaminated cell lines remains a common problem in cancer research, often leading to incorrect conclusions and irreproducible results (Souren et al., 2022). Short tandem repeat (STR) profiling is now widely regarded as the gold standard for cell line authentication, providing a genetic ‘fingerprint’ that confirms cell identity and prevents cross-contamination (Azari et al., 2007). However, although STR profiling ensures genetic consistency, it does detect broader phenotypic changes that occur in cell lines during extended culturing, a gap that is particularly relevant for laboratories that keep cell lines in culture beyond early passages.

The passage-dependent changes can be particularly relevant in advanced experimental models. Standard two-dimensional (2D) cell cultures, although convenient and widely used, are limited in their ability to replicate the complexity of the tumour microenvironment. Three-dimensional (3D) culture systems, such as spheroids, provide a more physiologically relevant platform for studying tumour biology. These systems better mimic the *in vivo* conditions by incorporating cell-cell and cell-matrix interactions. They allow for the study of tumour heterogeneity and drug responses in a setting that closely resembles the native tumour. Incorporating 3D cultures into preclinical research offers a significant advancement in the evaluation of therapeutic strategies and the understanding of tumour biology (Azari et al., 2007; Ben-David et al., 2018; Souren et al., 2022). Although 3D culture systems offer significant advantages, undetected clonal differences can compromise their reliability and lead to variable outcomes.

The SKOV3 cell line, derived from the ovarian tumour of a 64-year-old White female with adenocarcinoma, is one of the most often used models for studying ovarian cancer, with over 6500 pre-reviewed articles published in PubMed. Its ease of culture, well-documented characteristics and ability to form spheroids in 3D culture systems make it a preferred model for studying tumorigenesis, therapeutic efficacy and drug resistance in ovarian cancer (Hallas-Potts et al., 2019; Hasan et al., 2023; Weydert et al., 2020). Despite its widespread use, SKOV3 can also be subject to variability during prolonged culture, which can considerably affect experimental outcomes. In the Laboratory of Immunology at the National Cancer Institute of Lithuania, we documented an example of this phenomenon: two morphologically distinct SKOV3 strains (S1 and S2) were identified, both confirmed by STR to be SKOV3, yet displaying distinct morphologies, growth kinetics and drug responses. Although early-passage STR data were not available to pinpoint when the divergence began, our findings show how real-world culturing practices, often involving high passage numbers, can yield phenotypically different subpopulations.

Despite literature acknowledging cell line instability, few studies have systematically integrated STR authentication, phenotypic assays and transcriptomic profiling in both 2D and 3D models to quantify the molecular extent of clonal drift. This lack of integrated data leaves a critical gap in our understanding of how clonal heterogeneity impacts tumour modelling. To address the underappreciated risk of prolonged passaging-induced clonal evolution, we comparatively characterized SKOV3 S1 and S2 across morphology, growth kinetics, spheroid formation, surface-marker expression, drug sensitivities and transcriptomic signatures. This approach allowed us to delineate the extent of passage-induced divergence in an ovarian cancer model. Here, we show how phenotypic divergences can actively shape experimental outcomes, highlighting the need for more frequent and comprehensive characterization of widely used cancer cell lines to ensure reliable disease models.

## RESULTS

In this study, we investigated two morphologically distinct SKOV3 strains, referred to as S1 and S2. Surface marker expression analysis, drug sensitivity assays and RNA sequencing (RNA-seq) provided a detailed characterization of changes occurring up to passage 50 after isolation by differential trypsinization. All experiments were carried out following good culturing practices, with the strains seeded separately and maintained on the same shelf in the cell culture incubator.

### The STR profile of the SKOV3 cell strains reveals genetic instability

SKOV3 cell line strains S1 and S2 were observed over different passages, ranging from P2 to P50 after isolation by differential trypsinization. The cell strains exhibited distinct morphological differences that became more pronounced in later passages, grown in 2D cell culture conditions. We first decided to confirm the authenticity of strains by STR profiling, to ensure that phenotypic differences are due to subclonal drift rather than misidentification. To confirm the identity of the cancer cell line strains and to rule out any cross-contamination with other available cell lines, we performed STR analysis on morphologically distinct SKOV3 cell line strains after P25. DNA was extracted from these cell line strains, and the resulting STR profiles were compared. Although the STR profiles of the cultured cell line strains did not exactly match the original SKOV3 (HTB-77) cell line, they showed a high degree of similarity, with S1 and S2 both exhibiting a 0.96 match. Ultimately, both cell line strains at P25, while exhibiting morphological differences, were confirmed to be strains of the original SKOV3 (HTB-77) cell line.

STR profiling revealed genetic instability at the D5S818, vWA, D21S11, D18S51 and FGA loci. The SKOV3 S1 strain exhibited genetic instability at the vWA and FGA loci, whereas the SKOV3 S2 strain had genetic instability at the D5S818, D21S11 and D18S51 loci. In strain S1, the vWA locus, initially characterized by alleles 17 | 18, gained an additional allele 19, and the FGA locus, initially characterized by alleles 24 | 25 | 26, lost allele 25. In strain S2, the D5S818 locus, previously identified with alleles 11 | 11, lost an allele 11 and gained allele 10; the D21S11 locus, originally 30 | 31 | 31.2, gained allele 29; and the D18S51 locus, initially 16 | 17 | 18, lost allele 18 (Table 1).

**Table 1.** Short tandem repeat (STR) profiles

Locus	SKOV3 (HTB-77)	SKOV3 S1	SKOV3 S2
D5S818	**11 | 11**	**11 | 11**	**10 | 11**
D13S317	8 | 11	8 | 11	8 | 11
D7S820	13 | 14	13 | 14	13 | 14
D16S539	12 | 12	12 | 13	12 | 12
vWA	**17 | 18**	**17 | 18 | 19**	**17 | 18**
TH01	9 | 9.3	9 | 9.3	9 | 9.3
TPOX	8 | 11	8 | 11	8 | 11
CSF1PO	11 | 11	11 | 11	11 | 11
AMEL	X | X	X | X	X | X
D3S1358	14 | 14	14 | 14	14 | 14
D21S11	**30 | 31 | 31.2**	**30 | 31 | 31.2**	**29 | 30 | 31 | 31.2**
D18S51	**16 | 17 | 18**	**16 | 17 | 18**	**16 | 17**
Penta E	5 | 13	5 | 13	5 | 13
Penta D	12 | 13	12 | 13	12 | 13
D8S1179	14 | 15	14 | 15	14 | 15
FGA	**24 | 25 | 26**	**24 | 26**	**24 | 25 | 26**
D19S433	14 | 14.2	14 | 14.2	14 | 14.2
D2S1338	18 | 23	18 | 23	18 | 23

Profiles were compared between the SKOV3 (HTB-77) cell line and cell line strains S1 and S2 across 17 gene loci, including amelogenin (X and Y chromosomes). Genetic instability at the D5S818, vWA, D21S11, D18S51 and FGA loci was detected. Bold font indicates loci at which allelic changes were observed relative to the SKOV3 (HTB-77) profile. The similarity value between these cell line strains and the original SKOV3 cell line was 0.96, indicating a high degree of similarity.

### Morphological and functional characterization of SKOV3 cell strains

**Fig. 1.**
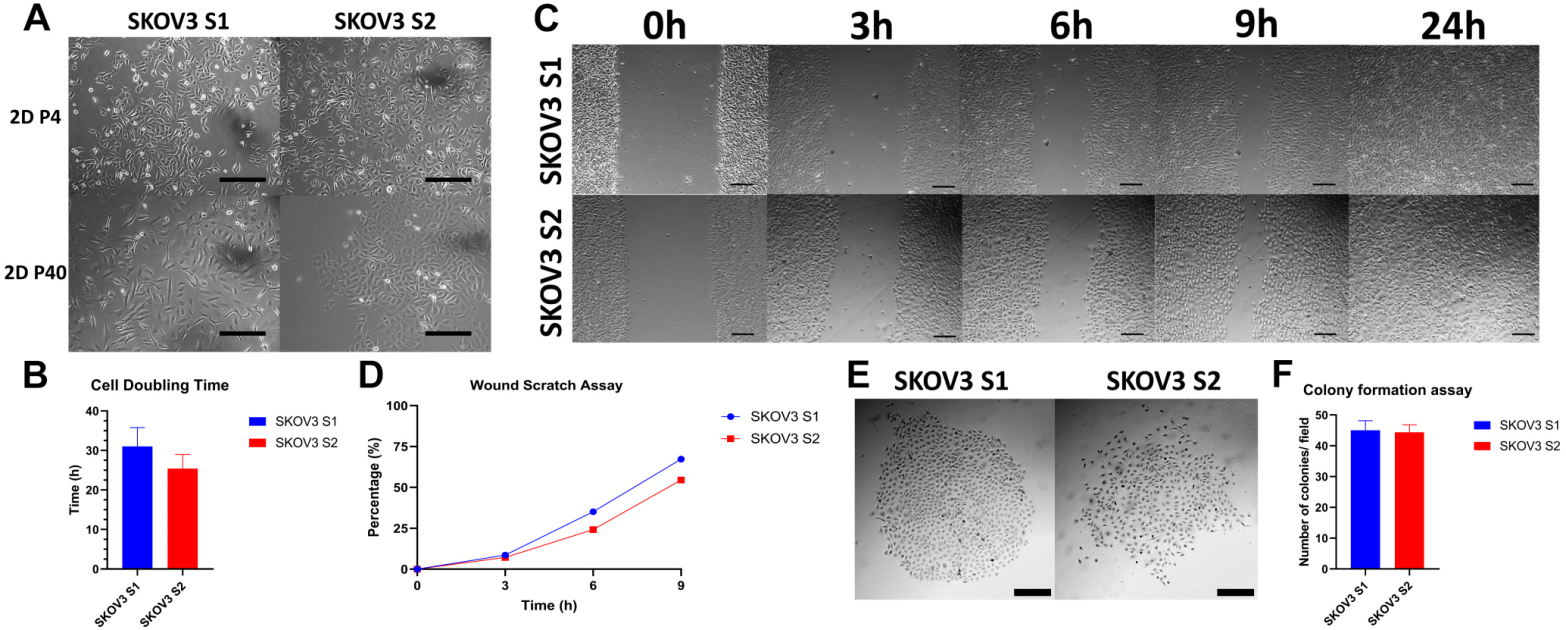
**SKOV3 S1 and S2 cell strains exhibit different cell morphology.** (A) Morphology of SKOV3 S1 and S2 at P4 and P40, grown in 2D cell culture. Bright-field images, magnification 100×, scale bars: 30 µm. (B) Cell strain doubling time, presented as mean±s.d., *N*=3 independent biological replicates; *n*=4-6 technical measurements per replicate. (C) Wound scratch assay of different SKOV3 cell strains. Bright-field images, magnification 40×, scale bars: 300 µm. (D) Wound scratch assay represented as percentages of wound closure over time, of *N*=3 biological replicates; *n*=3-4 scratches analysed per replicate. (E) Colony formation assay, showing colony morphology in bright-field images, magnification 40×, scale bars: 300 µm. (F) Colony formation assay, showing the number of colonies per field presented as mean±s.d., *N*=3 biological replicates; *n*=4 fields quantified per replicate.

SKOV3 cell line strains S1 and S2 were observed over different passages during cell culturing, from P2 to P50 after isolation by differential trypsinization. Strain S1 displayed a more elongated, spindle-shaped morphology and was slightly larger than strain S2. Cells of strain S1 grew more spread out, forming a uniform monolayer. In contrast, strain S2 appeared rounder, with irregular morphology, and the cells tended to cluster closely together (Fig. 1A). Notably, the elongated, mesenchymal-like shape of S1 versus the rounded clusters of S2 suggest that each strain may occupy a different phenotypic state [possibly relating to epithelial-mesenchymal transition (EMT) status]. Practically, such divergent growth patterns could influence how each strain interacts with its environment or responds to treatment. Therefore, we next aimed to evaluate the cell motility and clonogenic potential at a similar time point to when STR authentication was performed.

A wound scratch assay was performed to evaluate cell migration. Cell migration was observed up to 24 h. Both cell strains exhibited similar migration capabilities, with strain S2 showing slightly slower migration (Fig. 1D). However, no statistically significant difference was observed between the two strains when comparing cell migration ability (*P*=0.77). The wound was completely closed after 24 h (Fig. 1C). The doubling time of the cell strains was also counted (Fig. 1B). Strain S1 had a slightly longer doubling time than that of strain S2, with mean±s.d. values of 31.01±4.7 h for S1 strain and 25.42±3.5 h for S2 strain (*P*=0.15). Colony formation assays revealed no statistically significant differences between the two strains in terms of their ability to form colonies (*P*=0.78), with mean±s.d. values of 45±2.7 colonies for S1 strain and 44.4±2.2 colonies for S2 strain. However, the morphology of the colonies differed: strain S1 tended to form more even and symmetrical colonies, while strain S2 formed asymmetrical colonies with uneven edges (Fig. 1E,F).

### Differences between the SKOV3 strains in 3D cell culture formation

**Fig. 2.**
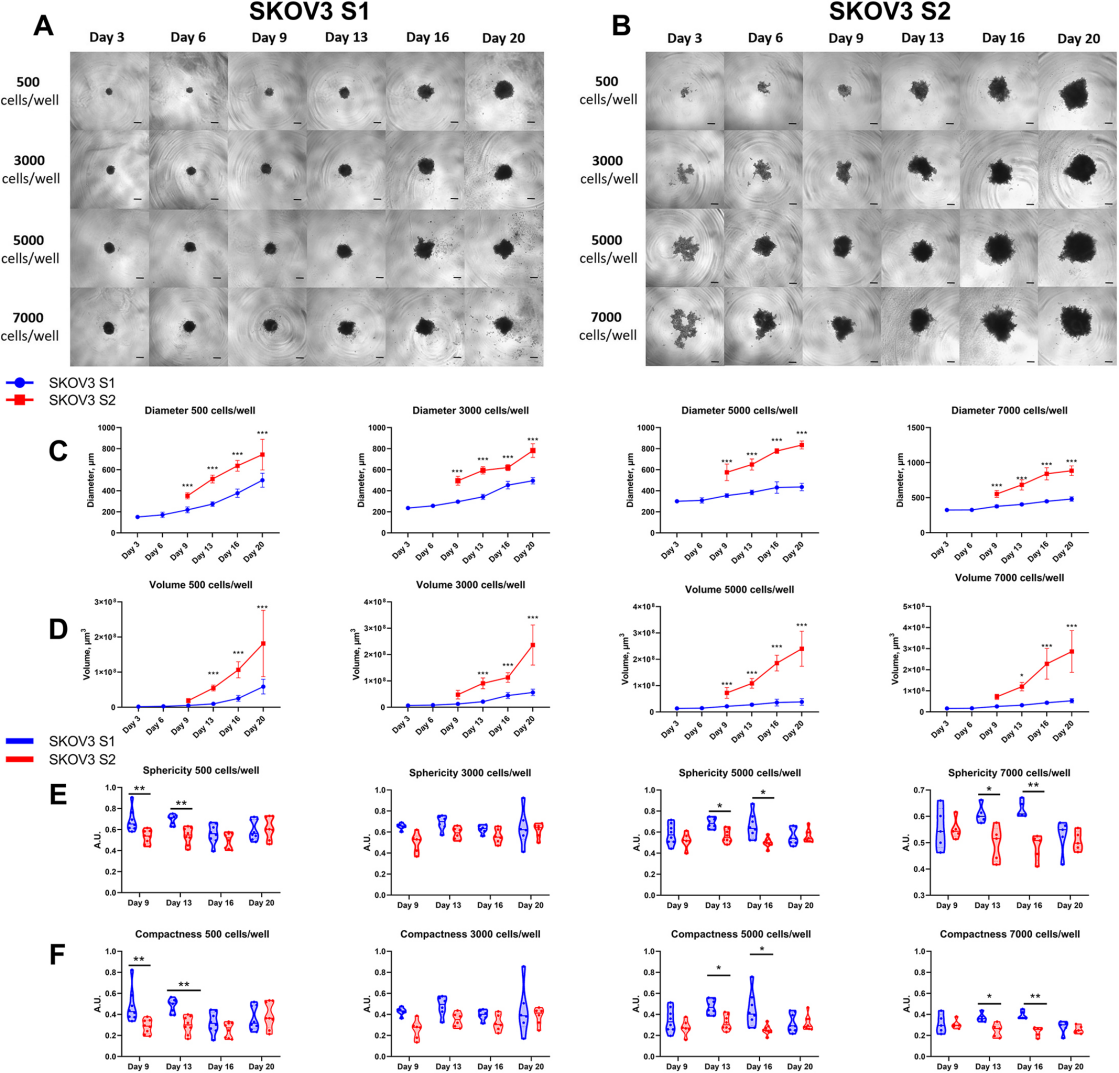
**3D cell culture, spheroid analysis comparing SKOV3 S1 and SKOV3 S2.** Spheroids [SKOV3 S1 (*n*=6-8) and SKOV3 S2 (*n*=6-8)] were generated from two independent biological replicates (*N*=2). (A) Bright-field images of SKOV3 S1 spheroids, magnification 40×. (B) Bright-field images of SKOV3 S2 spheroids, magnification 40×. For A and B, spheroids were seeded at various cell densities, ranging from 500 to 7000 cells per well, and imaged every 3 days; scale bars: 300 µm. (C) Diameter of S1 and S2 spheroids on different imaging days. ***P<0.001; two-way ANOVA followed by Tukey's multiple-comparison post-hoc test. (D) Volume of S1 and S2 spheroids on different imaging days. *P<0.05, ***P<0.001; two-way ANOVA followed by Tukey's multiple-comparison post-hoc test. (E) Sphericity of S1 and S2 spheroids. *P<0.05, **P<0.01; unpaired, two-tailed Student's *t*-test. (F) Compactness of S1 and S2 spheroids. *P<0.05, **P<0.01; unpaired, two-tailed Student's *t*-test. Diameter, volume, sphericity and compactness were estimated using AnaSP (v2.0) software (Piccinini et al., 2015). A.U., arbitrary units.

The ability of SKOV3 cell strains S1 and S2 to form 3D cell cultures was compared. All experiments were conducted between passages 20-30, after isolation by differential trypsinization. The cell line strains were seeded at different densities, ranging from 500 to 7000 cells per well. 3D cell culture growth was monitored over 20 days and imaged every 3 days (Fig. 2A,B). In SKOV3 S1, spheroid formation was observed as early as on day 3. Compact spheroids were consistently formed, regardless of the number of cells seeded per well. In contrast, the SKOV3 S2 strain only began to form 3D cell spheroids from day 6 to 9 depending on the seeding density. Better spheroid formation in the S2 strain was observed when the seeding density was 5000 or 7000 cells per well, with 3D cultures starting to become visible by day 6. The diameters, volumes, sphericity and compactness of spheroids formed by the S1 and S2 were analysed and compared (Fig. 2C-F). For the S1 strain, spheroids were measured from day 3, when the spheroids first became visible. Measurements for the S2 strain began only on day 9, once the spheroids were well defined. Both strains exhibited 3D cell culture growth, reaching their maximum spheroid size, thereby confirming cell viability. However, S1 spheroids grew more slowly than S2 spheroids. When comparing spheroid diameters (Fig. 2C), it was visibly and statistically confirmed that the S2 strain formed spheroids with a larger diameter than that of spheroids formed by the S1 strain. The same trend was observed when comparing spheroid volumes, with S2 3D cultures being significantly larger in volume than S1 3D cultures (Fig. 2D). After analysing spheroid compactness and sphericity from day 9 onward (Fig. 2E,F), it was observed that S1 spheroids were consistently more spherical and compact than S2 spheroids. As a result, this analysis suggests that the smaller size of S1 spheroids is due to their better compactness, which results in smaller spheroid diameters and volumes. However, the S2 strain, with poorer ability to form 3D cell cultures along with lower compactness, produced larger spheroids. This difference in 3D growth demonstrates how clonal divergence within the same cell line can affect the quality and timing of spheroid formation, a critical aspect of modelling the tumour microenvironment.

### SKOV3 cell strains exhibit different surface phenotypes at various passages

**Fig. 3.**
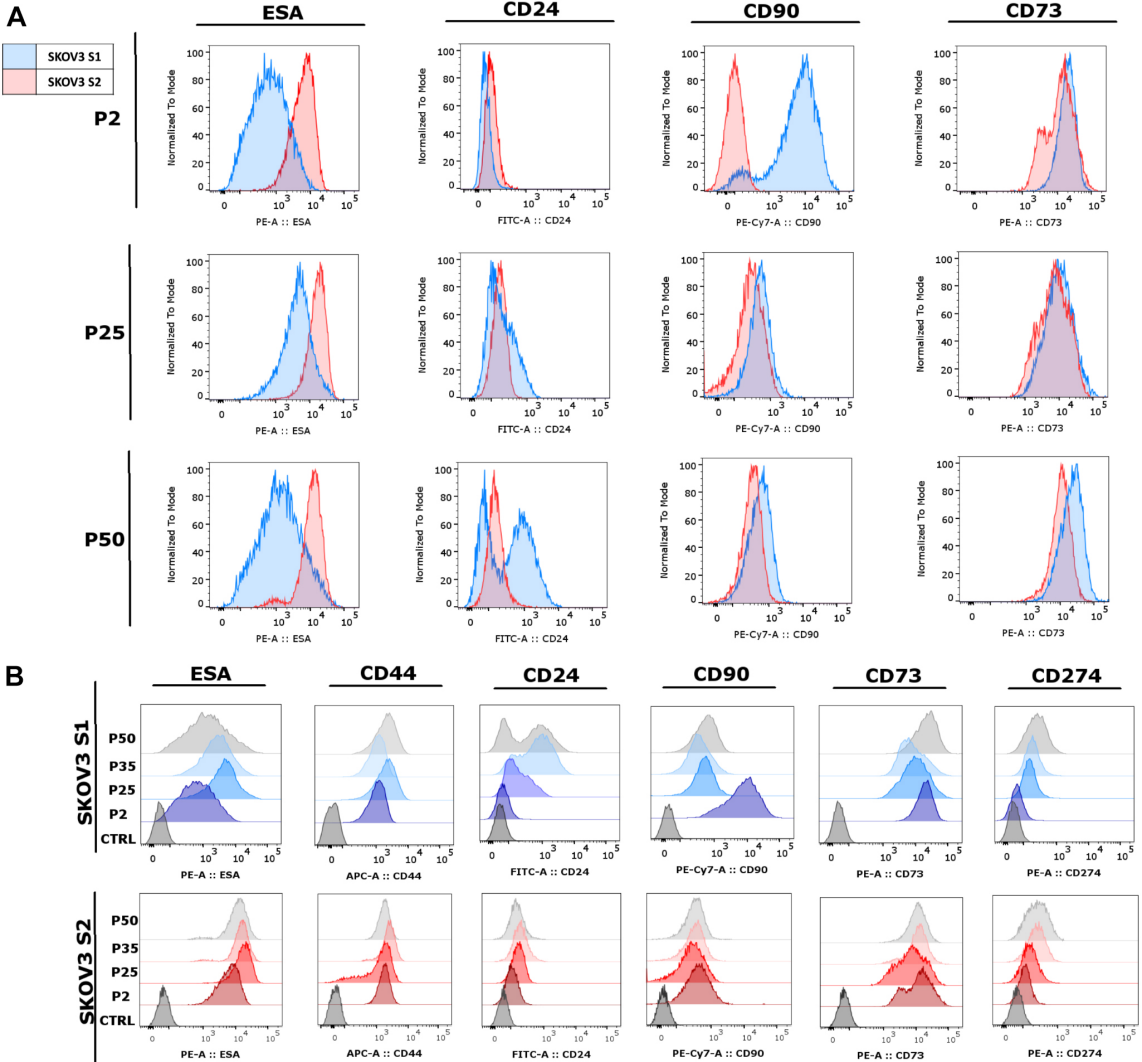
**Flow cytometry analysis of SKOV3 strains S1 and S2 was performed at different passages, from P2 to P50 after isolation by differential trypsinization.** (A) Histograms display the expression of markers ESA, CD24, CD90 and CD73 at passages 5, 25 and 50. (B) Changes in marker expression are shown in histograms for passages 2, 25, 35 and 50. The markers analysed include ESA, CD44, CD24, CD90, CD73 and CD274.

SKOV3 strains S1 and S2 were analysed by flow cytometry from P2 to P50 after isolation by differential trypsinization, focusing on markers related to epithelial cell properties, cell adhesion, immune system evasion and cancer stemness. The epithelial markers included epithelial specific antigen (ESA; also known as EPCAM) and CD44, a glycoprotein involved in cell adhesion, migration, and commonly associated with cancer stemness (Baeuerle and Gires, 2007; Zöller, 2011). The analysis also measured cancer stemness markers: CD24, associated with cell adhesion and metastatic potential (Altevogt et al., 2021); CD90 (also known as THY1), a glycoprotein linked to tumorigenicity (Sauzay et al., 2019); CD73 (also known as NT5E), an enzyme involved in immune suppression within the tumour microenvironment (Roh et al., 2020); and CD274 (also known as PD-L1), which helps tumours evade immune detection (Cha et al., 2019). Notable differences were observed in the expression of markers ESA, CD24, CD90 and CD73 between the SKOV3 cell strains (Fig. 3A). Both strains expressed the ESA marker, but with higher expression levels observed in the S2 strain. ESA expression remained stable within each strain across P25 to P50 (Fig. 3B). Neither strain expressed CD24 in the early passages to P2. However, in later passages, the S1 strain began to express CD24, leading to the formation of two distinct cell populations – one with CD24 expression and one without. In contrast, the S2 strain consistently did not express CD24, remaining stable across all passages. CD90 was initially expressed at higher levels in the S1 strain at P2. As the passages progressed, its expression in S1 decreased and became similar between the two SKOV3 strains at P25 and P50. CD73 was expressed at similar levels between the strains and remained stable between the passages.

Analysis across passages (Fig. 3B) revealed that marker expression varies between cell strains and can change across different passages. Although some markers, such as CD44 and CD73, remained relatively stable and consistent over time, others, such as CD274, gradually increased in expression in later passages or began to be expressed, such as CD24 in the S1 strain in later passages. Overall, the altered expression of markers linked to stemness, adhesion and immune evasion suggests functional divergence between the strains. Such variation implies that using one strain over the other could lead to different results in assays for cancer stem cell properties or immunotherapy targets.

### SKOV3 strains show different response to chemotherapeutic drugs

**Fig. 4.**
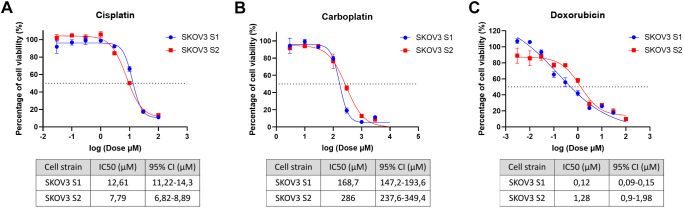
**Drug sensitivity analysis of SKOV3 S1 and S2.** The CCK-8 values obtained from treated SKOV3 cell strains were normalized to those of untreated samples. (A) SKOV3 S2 is more sensitive to cisplatin (IC50 of 7.79 μM compared to 12.61 μM in S1). (B) SKOV3 S1 shows greater sensitivity to carboplatin (IC50 of 168.7 μM compared to 286 μM in S2). (C) SKOV3 S1 is more sensitive to doxorubicin (IC50 of 0.12 μM compared to 1.28 μM in S2). The data are from two independent biological replicates (*N*=2), presented as mean±s.d. [for each biological replicate (*N*), *n*=3 technical replicates were analysed per drug concentration and averaged within that *N*]. The dashed lines indicate a 50% reduction in cell viability.

We tested SKOV3 cell strains S1 and S2 with three chemotherapy drugs: cisplatin and carboplatin, which are platinum-based drugs that cause DNA crosslinks, and doxorubicin, which intercalates into DNA, inhibits topoisomerase II and generates free radicals, leading to DNA damage (Dasari and Tchounwou, 2014; Rivankar, 2014). The CCK-8 assay was used to assess the cytotoxicity of these drugs in the SKOV3 S1 and S2 strains and to determine their half-maximal inhibitory concentration (IC50) values. For this analysis, SKOV3 strains between passages 30 and 40 were used, and IC50 values were determined after 48 h of drug exposure. As shown in Fig. 4, strain S1 showed higher (*P*=0.0001) sensitivity to carboplatin, with an IC50 of 168.7 µM [95% confidence interval (CI): 147.2-193.6 μM], and doxorubicin, with an IC50 of 0.12 µM (95% CI: 0.09-0.15 μM), than strain S2, which had IC50 values of 286 µM (95% CI: 237.6-349.4 μM) for carboplatin and 1.28 µM (95% CI: 0.9-1.98 μM) for doxorubicin (Fig. 4B,C). In contrast, strain S2 was more sensitive (*P*=0.0001) to cisplatin, with an IC50 of 7.79 µM (95% CI: 6.82-8.89 μM), whereas strain S1 had an IC50 of 12.61 µM (95% CI: 11.22-14.3 μM) (Fig. 4A). These results show that the two SKOV3 cell strains, S1 and S2, respond differently to specific chemotherapeutic drugs, indicating the impact of prolonged culture on cell drug sensitivity. In practical terms, a compound demonstrating efficacy in one strain (S1) may exhibit substantially reduced activity in another (S2), underscoring how clonal evolution during cell culture can confound drug screening outcomes.

### Transcriptomic analysis indicates strain-specific gene expression profiles

**Fig. 5.**
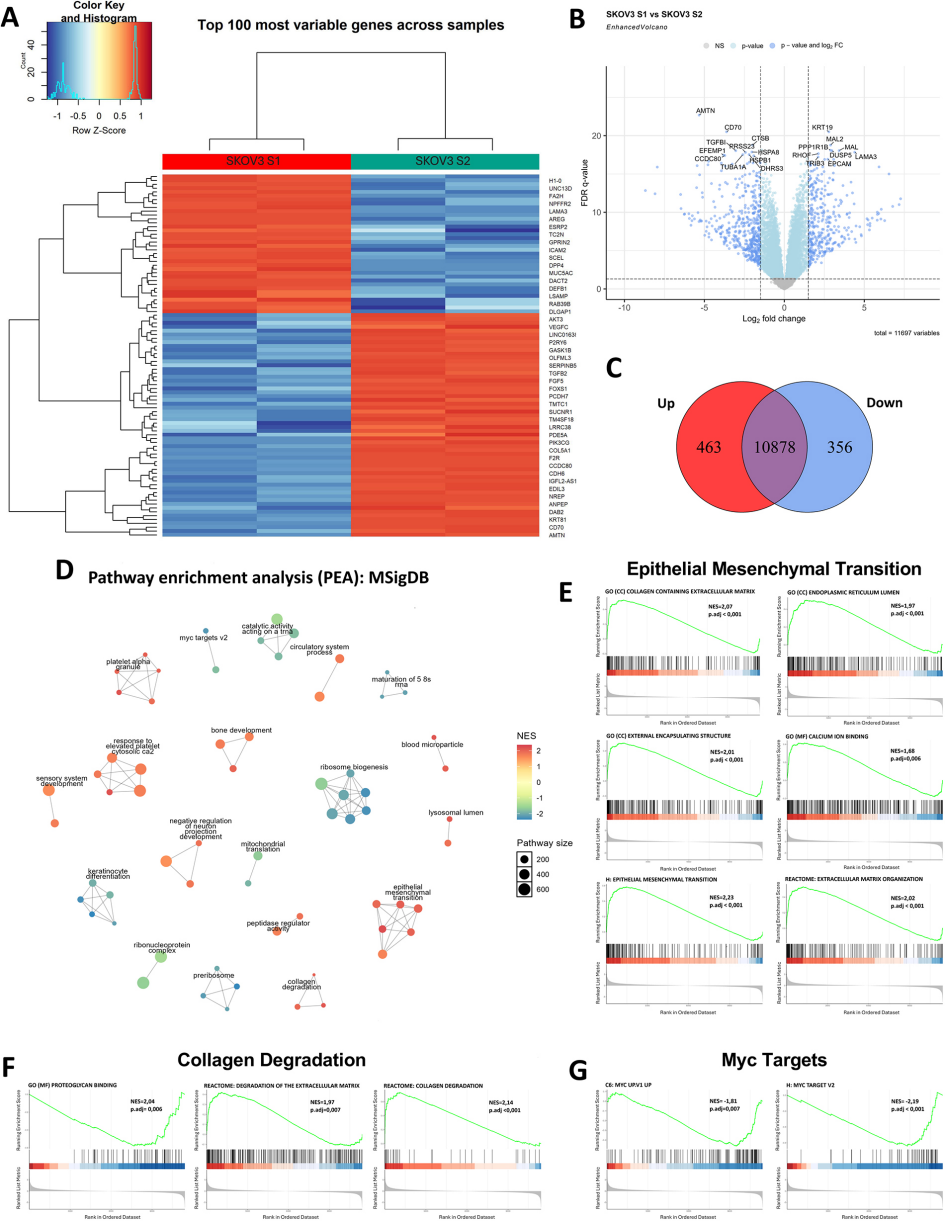
**Transcriptomic analysis of strain-specific genes using bulk RNA sequencing (RNA-seq).** Data are from two independent biological replicates (*N*=2). (A) Shared and unique gene expression, with 10,878 shared genes, 463 upregulated and 356 downregulated in SKOV3 S1 compared to S2. (B) Differentially expressed genes (DEGs) between SKOV3 S1 and S2. Log2 fold changes and false discovery rate (FDR)-adjusted *P*-values indicate significant strain-dependent gene expression changes. FC, fold change; NS, not significant. (C) The top 100 most variable genes show clear clustering of SKOV3 S1 and S2, indicating distinct transcriptional profiles. (D) Pathway enrichment analysis – including MSigDB database hallmark pathways (H) *N*=50; Gene Ontology (GO) biological processes (C5: GO:BP) *N*=7608, cellular components (C5: GO:CC) *N*=1026 and molecular functions (C5: GO:MF) *N*=1820; Kyoto Encyclopedia of Genes and Genomes (KEGG) pathways (C2: KEGG) *N*=658; Reactome pathways (C2: CP:REACTOME) *N*=1736, WikiPathways (C2: CP:WIKIPATHWAYS) *N*=830; and oncogenic signatures (C6) *N*=189 – demonstrates enrichment of epithelial-mesenchymal transition (EMT)-related pathways and collagen degradation in SKOV3 S1, while MYC-regulated pathways are enriched in SKOV3 S2. (E) Gene set enrichment analysis (GSEA) of EMT-related pathways: ‘collagen containing extracellular matrix’, ‘endoplasmic reticulum lumen’, ‘extracellular encapsulating structure’, ‘calcium ion binding’, ‘epithelial mesenchymal transition’ and ‘extracellular matrix organization’. NES, normalized enrichment score; p.adj, adjusted *P*-value. (F) GSEA of collagen degradation pathways: ‘proteoglycan binding’, ‘degradation of extracellular matrix’ and ‘collagen degradation’. (G) GSEA of MYC target pathways, including ‘MYC up.V1 up’ and ‘MYC targets V2’.

To investigate the transcriptional differences between the two SKOV3 strains, S1 and S2, in 2D culture, bulk RNA-seq was performed at passage P25 after isolation by differential trypsinization. Differential gene expression analysis identified 463 upregulated and 356 downregulated genes in SKOV3 S1 in comparison to SKOV3 S2. Of a total of 11,697 expressed genes, 10,878 genes were shared between the two strains with no significant expression difference (Fig. 5A,B). The top 100 most variable genes between SKOV3 S1 and S2 (Fig. 5C) showed clear segregation of the two strains into distinct transcriptional clusters (full gene list in Table S1). This clustering shows significant differences in gene expression patterns, which may regulate the phenotypic differences observed between the strains.

Pathway enrichment analysis was performed to investigate the functional connections associated with the distinct gene expression profiles observed in SKOV3 S1 and S2 strains (Fig. 5D). In SKOV3 S1, pathways associated with EMT and extracellular matrix remodelling, including extracellular matrix organization and collagen degradation, were significantly upregulated (Fig. 5E,F). These findings indicate that SKOV3 S1 displays enhanced mesenchymal characteristics, and a higher invasive potential, than SKOV3 S2. The enrichment of collagen degradation pathways suggests increased extracellular matrix remodelling, which is critical for tumour cell migration and invasion. In contrast, MYC-regulated pathways, which are associated with cell proliferation and metabolic reprogramming, were downregulated in SKOV3 S1 (Fig. 5G). This downregulation of MYC activity aligns with a potentially slower proliferative phenotype in this strain despite its invasive potential. SKOV3 S2 exhibited significant downregulation of EMT-related pathways and collagen degradation compared to SKOV3 S1, suggesting a less mesenchymal and less invasive phenotype (Fig. 5E,F). Instead, SKOV3 S2 demonstrated upregulation of MYC-regulated pathways (Fig. 5G), which are crucial for driving cell proliferation and metabolic activity. The increased MYC activity in SKOV3 S2 aligns with enhanced growth dynamics, indicating that SKOV3 S2 relies more on proliferative and metabolic reprogramming rather than invasive processes. Overall, these findings show the molecular and functional adaptations of SKOV3 strains under prolonged culture conditions, revealing different possible strategies for tumour progression.

### Transcriptomic analysis of SKOV3 strains in 2D and 3D cultures reveal differences in gene expression

**Fig. 6.**
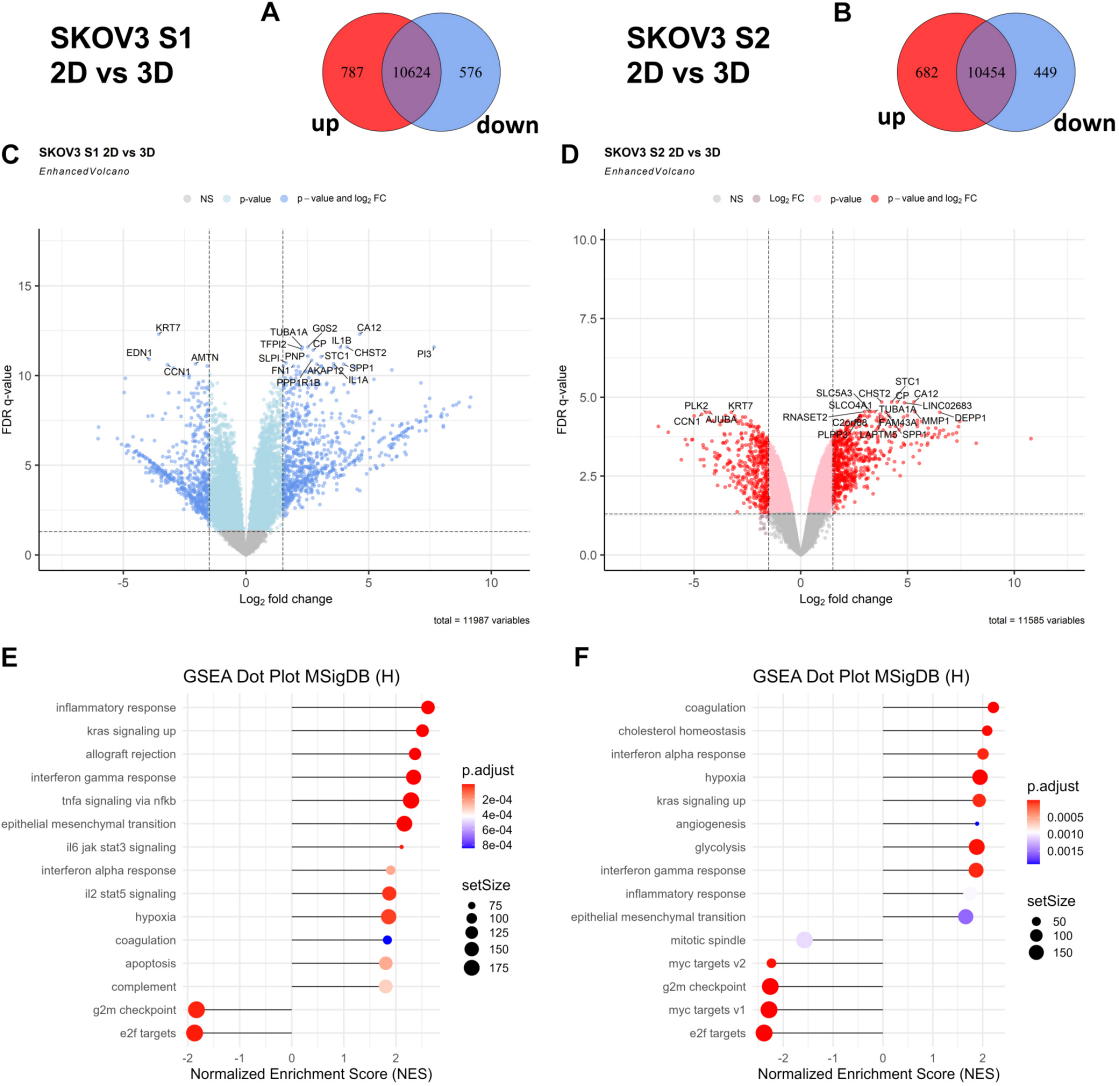
**Transcriptomic analysis between 2D and 3D culture conditions for SKOV3 S1 and S2 strains using bulk RNA-seq.** Data are from two independent biological replicates (*N*=2). (A,B) Venn diagrams of DEGs between 2D and 3D cultures for SKOV3 S1 (A) and SKOV3 S2 (B) strains. (C,D) Volcano plots of DEGs in SKOV3 S1 (C) and SKOV3 S2 (D) cultured in 2D versus 3D systems. Log2 fold changes and FDR-adjusted *P*-values indicate significant culture-dependent gene expression changes. (E,F) GSEA of MSigDB hallmark pathways shows distinct pathway enrichment between SKOV3 S1 (E) and SKOV3 S2 (F).

To address how different cancer cell strains can influence the transcriptome of the spheroid, we also analysed transcriptional differences between SKOV3 S1 and S2 strains under 2D and 3D culture conditions. Bulk RNA-seq was performed at passage P25, after isolation by differential trypsinization, to analyse these changes. In SKOV3 S1, 787 genes were upregulated, and 576 genes were downregulated, in 3D culture compared to 2D, with 10,624 genes shared between different culturing conditions (Fig. 6A). In SKOV3 S2 strain, 682 genes were upregulated, and 449 genes were downregulated, with 10,454 genes shared between the 3D and 2D conditions (Fig. 6B). The upregulated genes of SKOV3 S1 in 3D culture included *FN1*, *TUBA1A*, *IL1B* and *SPP1* and downregulated genes included *KRT7* and *EDN1* (Fig. 6C). In contrast, SKOV3 S2 exhibited upregulation of genes *PLK2*, *CCN1* and *KRT77*, with *MMP1* and *SPP1* genes downregulated under 3D conditions (Fig. 6D).

Gene set enrichment analysis (GSEA) provided further insights into the functional differences between the two strains under 3D culture conditions. In SKOV3 S1, pathways associated with inflammatory response, TNF-α signalling via NF-κB, and EMT were significantly enriched (Fig. 6E). Collagen degradation and hypoxia-related pathways were also upregulated. These findings suggest that SKOV3 S1 grown in 3D influences EMT and mesenchymal features to enhance its invasive capacity. In contrast, SKOV3 S2 grown in 3D exhibited significant enrichment in pathways related to glycolysis and angiogenesis (Fig. 6F), reflecting a metabolic and proliferative response. Downregulated MYC targets and mitotic spindle pathways in SKOV3 S2 were also visible.

Notably, several pathways were uniquely enriched in the 3D culture conditions, showing the influence of dimensionality on cancer cells. Hypoxia-related pathways were strongly activated in both SKOV3 S1 and S2, reflecting the oxygen-limited conditions characteristic of the 3D culturing. In SKOV3 S1, pathways associated with coagulation and extracellular matrix organization were enriched, emphasizing its adaptation to the complex mechanical and biochemical environment in 3D culture. In SKOV3 S2, angiogenesis and cholesterol homeostasis pathways were uniquely enriched, showing its metabolic adaptation and reliance on vascular-like features for growth in 3D conditions. These potentially distinct 3D-enriched pathways illustrate the physiological relevance of 3D models for recapitulating *in vivo* tumour conditions. To conclude, these results reveal distinct transcriptional and functional profiles for SKOV3 S1 and S2 under 3D culture conditions, highlighting that subclonal differences can substantially alter outcomes even in more physiologically relevant models.

## DISCUSSION

Our study reveals that a commonly used ovarian cancer cell line, SKOV3, can split into distinct subclonal strains with divergent phenotypes during routine culture, a phenomenon with serious implications for cancer model reliability. The resulting SKOV3 cell strains, S1 and S2, were authenticated using STR profiling and confirmed to match SKOV3 (HTB-77), despite exhibiting different morphologies and molecular profiles. STR profiling fulfils the purpose of authentication testing, as defined by The International Cell Line Authentication Committee (ICLAC) (ICLAC Terms of Reference Version 5.0). However, standard authentication methods, such as STR, do not consider the genetic and functional diversity within a cell line, which can significantly compromise reproducibility across experiments or laboratories (Ben-David et al., 2018; Kleensang et al., 2016). Our findings highlighted the differences between the strains, including cell surface marker expression, 3D spheroid formation, transcriptomic profiles and sensitivity to chemotherapeutic drugs. These differences reflect the impact of clonal evolution in long-term culture – an issue that is well known but is rarely assessed using multiple endpoints in a single study. This SKOV3 case is likely not unique – similar variation within the same cell lines has also been observed and described in other cancer cell lines, such as MCF-7, MDA-MB-231, Ishikawa and HeLa, raising critical questions about the credibility of commonly used cancer models (Ben-David et al., 2019; Kasai et al., 2016; Khan et al., 2017; Kleensang et al., 2016; Liu et al., 2019). Our study systematically correlated genotypic drift (STR instability) with broad phenotypic consequences (morphology, 3D growth, markers, drug response, transcriptomics) within the same model, emphasizing that additional characterization methods are essential for ensuring experimental reliability, especially because routine tests such as STR profiling overlook the subtle subclonal drift that may emerge during prolonged passaging.

Recommended passage numbers vary slightly across studies. A mini-review on passage-related drift in cancer cell lines shows that shifts in gene expression, copy number and drug response build up after ∼20 passages and therefore advises restarting from a low-passage cell culture at that point (Prasad et al., 2023). Good Cell Culture Practice guidelines describe this as well, noting that maintaining a culture for more than ∼10 passages, or keeping cells in the culture for more than 3 months, risks genetic and phenotypic drift unless the cell line is re-authenticated for the specific assay (Geraghty et al., 2014). Laboratory data also confirm, as Handl et al. (2020) tracked HK-2 kidney cells from passage 3 to 15 and found that their sensitivity to cisplatin and cadmium changed between the passages even though growth rates remained stable. Taken together, these literature sources support a practical rule: perform key experiments, including drug toxicity assays, within the first 5-20 passages after thawing, and return to an early-passage cell culture once that limit is reached to keep experimental results reproducible.

In the commonly described characteristics of the SKOV3 cell line, these cells typically exhibit epithelial-like morphology, reflective of their origin from ovarian epithelial adenocarcinoma (Beaufort et al., 2014). However, in our study, strain S1 of SKOV3 displayed a spindle-shaped, mesenchymal-like morphology, and strain S2 appeared rounder and less defined, suggesting phenotypic variation from the widely used description of SKOV3 cells. Transcriptomics analysis further supported these findings, with upregulation of EMT- and extracellular matrix-related pathways in S1, whereas S2 showed enrichment in MYC-regulated pathways related to proliferation and metabolism.

Even though bulk RNA-seq data indicate upregulation of EMT in S1, this type of data alone cannot determine whether the cells are in a complete EMT state or in an intermediate (partial-EMT) state. Differentiating between complete and partial EMT requires single-cell approaches combined with more detailed characterization (Pastushenko et al., 2018; Subhadarshini et al., 2023). Therefore, we interpret the enrichment of EMT in S1 as evidence of movement along the epithelial-mesenchymal axis, but we cannot specify the exact stage of transition, and we acknowledge this limitation in the present study. Further phenotypic differences between the S1 and S2 strains were particularly evident in 3D culture. S1 cells formed compact and spherical spheroids within 3 days, whereas S2 cells formed larger, less compact spheroids requiring higher seeding densities. Comparing our results to published findings, variation in the SKOV3 cell line strains is evident, particularly regarding the S2 strain, as the SKOV3 cell line is reported as one of the ovarian cell lines that forms compact spheroids in 3D culture, visible by day 3 (Chaundler et al., 2023 preprint; Myungjin Lee et al., 2013). These results highlight how incorporating 3D models can accentuate functional distinctions, giving a more physiologically relevant view of clonal heterogeneity.

Surface marker expression highly differed from already published scientific studies. Literature describes SKOV3 as having high expression of CD44 and CD73, weaker expression of CD24, and very little or no expression of ESA (Beaufort et al., 2014; Roh et al., 2020). The expression levels of CD90 and CD274 remain unclear in the SKOV3 cell line. In our study, both cell strains expressed high levels of ESA, CD44, CD73 and CD90. The SKOV3 S1 strain expressed CD24 in later passages, whereas S2 did not express it at all, and both strains exhibited very weak expression of CD274. These differences could result from clonal evolution, culture conditions and passage number, as extended *in vitro* passaging can select subpopulations with distinct molecular profiles. Importantly, such divergence in key surface markers can profoundly influence experiments that rely on immunophenotyping or targeted therapies, and it highlights the necessity of more routine checks beyond simple identity confirmation.

Drug toxicity assays also revealed differences between the strains. The S1 strain showed ten times higher (0.12 µM) sensitivity to doxorubicin than the S2 (1.28 µM) strain. In the literature, SKOV3 cell line IC50 values for doxorubicin range from 0.03 to 0.15 µM, according to the Genomics of Drug Sensitivity in Cancer (GDSC) database, and to 0.34 µM, as shown in other studies (Ittiudomrak et al., 2019; Yang et al., 2013). This variability in therapeutic responses aligns with findings from a study by Uri Ben-David and colleagues, which indicate that clonal heterogeneity in cell lines, such as MCF-7, can compromise the reproducibility of drug toxicity studies (Ben-David et al., 2018). These results highlight the importance of understanding cell line evolution *in vitro* when interpreting drug sensitivity and resistance, as different cell strains may have varying responses to the same drug. The reproducibility of drug-response assays still remains a critical challenge in cancer research, as demonstrated in the comprehensive study conducted by Niepel et al. (2019) under the National Institutes of Health Library of Integrated Network-Based Cellular Signatures Program, with the possible causes discussed, such as cell clonal variation or genetic drift. In conclusion, this cell line strain variability has profound consequences for preclinical drug testing, particularly where consistent and reliable results are essential for translating laboratory findings into effective design of clinical therapies.

A comparison of 2D and 3D culture models of SKOV3 S1 and S2 revealed significant transcriptomic differences, reflecting the distinct culturing conditions provided by each system. In the 3D spheroid culture, cells from both S1 and S2 strains showed upregulation of biological pathways associated with hypoxia, EMT and angiogenesis. These results align with the structural and physiological conditions typically found in the tumours. These results are also consistent with prior studies indicating that 3D cultures better mimic *in vivo* tumour conditions than do 2D monolayer cultures (Sharma et al., 2024). Transcriptomic differences between the models showed the importance of selecting an appropriate culture system to study specific biological processes, as the 2D model fails to replicate the complex gene expression dynamics observed in 3D cultures. When considered alongside the strain-specific differences, these results show that the interplay between culture dimensionality and subclonal divergence can significantly shape experimental outcomes as well.

For SKOV3 cells, as for other widely used cancer cell lines, additional characterization beyond standard authentication is essential to ensure consistency and reliability of experimental results. Without such measures, the effectiveness of these models in advancing cancer research remains limited. Furthermore, these finding highlight the need for additional characterization methods, such as next-generation sequencing, and comparative genomic hybridization to detect and account for clonal evolution and genetic variability (Gisselsson et al., 2019). Integrating functional assays with transcriptomic profiling, as shown in this study, offers a strong framework for understanding and addressing the complexities of cell line heterogeneity. However, this study, while providing significant insights into the variability of SKOV3 cell strains, encounters certain limitations that could impact the broader application of its findings. The analysis was restricted to two strains, potentially limiting a broader understanding of variability across different cell lines. In addition, RNA-seq was performed with *N*=2 biological replicates per condition, which we acknowledge as a limitation of the transcriptomic analyses. The focus was also primarily on specific genetic markers and pathways, which may not capture the full spectrum of genetic alterations occurring during cell line evolution. Nonetheless, these findings raise critical considerations for researchers using SKOV3 as a model system. The observed phenotypic plasticity suggests that SKOV3, although widely used, may not always be a stable model for ovarian cancer research. Whether such plasticity is reversible remains an open question, encouraging further studies on the environmental and genetic factors influencing cellular transitions. These findings also emphasize the need for stricter quality control in cell culture practices. Integrating advanced genomic techniques and functional assays with existing authentication protocols is essential to address clonal evolution challenges effectively. Additionally, detailed methodological descriptions, including information on cell passages, authentication methods such as STR profiling and other characterizations, are crucial to ensure experimental reproducibility and the validity of *in vitro* cancer models.

Although this study characterizes two phenotypically divergent SKOV3 strains, we do not regard either as the more representative or biologically relevant model. Rather, our findings highlight the practical challenge that such phenotypic variability can arise and persist undetected during routine passaging. The decision to use S1, S2 or a mixed population should be guided by the biological question under investigation, rather than by anticipated results. In practice, laboratories may wish to establish a minimal characterization checklist, such as routine morphology review, STR confirmation, and a small panel of functional or drug-sensitivity assays, tailored to the endpoints of each study. These observations show the importance of regularly monitoring morphological features, maintaining detailed records of passage history and recognising the potential impact of subclonal drift on assay outcomes, particularly in long-term cultures.

In summary, by assessing multiple endpoints, including surface marker expression, chemotherapeutic response and transcriptomic profiles in 2D and 3D culture, we show that, upon prolonged passaging, even a well-established cell line like SKOV3 can diverge considerably despite retaining a validated genetic signature. These observations highlight that passage history extends beyond mere technical details, as it can profoundly influence experimental outcomes and translational interpretations. By integrating expanded multi-omics analyses and rigorously documenting cell-line passage history, researchers can minimize the confounding influence of subclonal evolution and thereby strengthen the reproducibility of preclinical research.

## MATERIALS AND METHODS

### Cell culture and reagents

The original SKOV3 cell line was received from American Type Culture Collection (ATCC^®^; cat. no. HTB–77™, Lot 70008371; Certificate of Analysis reference date 16 October 2017) at passage 24. Cells were cultured for at least additional 30 passages under identical conditions, at which point two morphologically distinct subpopulations became apparent and were observed to coexist within the same culture. Following the protocol described in Morata-Tarifa et al. (2016), these subpopulations were isolated by differential trypsinization based on their detachment kinetics, with S1 taking longer to detach than S2, and were subsequently maintained as separate strains, named S1 and S2, with passages counted from this point as P0.

Both S1 and S2 strains were cultured in Dulbecco's modified Eagle medium (DMEM; Gibco™, cat. no. 11965092) supplemented with 10% fetal bovine serum (FBS; Gibco™, cat. no. 16140071) and 1% penicillin-streptomycin (PS; Gibco™, cat. no. 15140122). Cells were maintained at 37°C in a humidified atmosphere of 5% CO_2_ and passaged at ∼80% confluence using 0.25% Trypsin–EDTA (Gibco™, cat. no. 25200056). Master and working cell banks were generated by cryopreserving cells every three to four passages in freezing medium (90% FBS, 10% DMSO) stored in −80°C. All experiments used cells between passages 2 and 50 after isolation by differential trypsinization. Cell viability was routinely confirmed by Trypan Blue (Gibco™, cat. no. 15250061) exclusion.

### STR profiling

Genomic DNA was extracted using a QIAamp DNA Micro Kit (50) (Qiagen, cat. no. 56304) according to the manufacturer's protocol. Seventeen STR loci (D5S818, D21S11, D7S820, CSF1PO, D2S1338, D3S1358, vWA, D8S1179, D16S539, TPOX, TH01, D19S433, D18S51, FGA, D13S317, Penta E and Penta D) along with the Amelogenin sex-determining marker were amplified and analysed by capillary electrophoresis. STR profiles were compared using the Cellosaurus STR Similarity Search Tool (CLASTR 1.4.4). The similarity value was calculated using the Tanabe algorithm.

### Morphological evaluation

Morphological differences between SKOV3 S1 and SKOV3 S2 strains were assessed using phase-contrast microscopy. Cells were cultured under standard culturing conditions and imaged between passage 2 and 50 using an inverted phase-contrast microscope (OPTIKA ITALI IM-5) at 100× magnification. Cell shape, size and other morphological features were documented.

### Doubling time calculation

To determine the doubling time, cells were seeded at an initial density of 2×10^5^ cells per well in 12-well plates. Cells were harvested and counted after 48 h using a haemocytometer and Trypan Blue exclusion assay. Cell doubling time was calculated using the following formula:

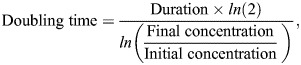
where *ln* represents the natural logarithm (base e).

### Wound scratch assay

Cell migration was analysed using the wound scratch assay. SKOV3 S1 and SKOV3 S2 cells were seeded in 12-well plates at a density of 5×10^5^ cells per well and cultured for 24 h, until a confluent monolayer was formed. A sterile 200 µl pipette tip was used to create a straight scratch in the monolayer. The wells were washed twice with phosphate-buffered saline (PBS; Gibco™, cat. no. 10010023) to remove detached cells and debris, followed by the addition of complete DMEM. Images of the scratch area were captured immediately (0 h) and at 3, 9, 6 and 24 h using an inverted microscope at 40× magnification. The images were analysed, and wound closure (%) was calculated using Wound Healing Size Tool, an ImageJ/Fiji software plugin (Suarez-Arnedo et al., 2020).

### Colony formation assay

The colony-forming ability of cell strains was evaluated by seeding 50 cells per well in six-well plates. Cells were cultured for 14 days in complete DMEM, with medium changes every 4 days. At the end of the incubation period, colonies were fixed with 90% ethanol for 10 min and stained with 0.1% Crystal Violet solution for 10 min. Colonies containing ≥50 cells were counted manually under a light microscope.

### Spheroid formation

Spheroid formation was evaluated by seeding S1 and S2 cell strains in UltraPure™ agarose (Invitrogen™, cat. no. 16500500)-covered U-bottom 96-well plates, prepared following a protocol from Friedrich et al. (2009). Cells were seeded at densities ranging from 500 to 7000 cells per well, with 200 µl DMEM supplemented with 10% FBS and 1% PS. The 3D cell cultures were maintained at 37°C in a humidified atmosphere with 5% CO_2_ for 20 days. Cell cultures were observed and imaged every 3 days using an inverted microscope. Image analysis was performed using AnaSP (v2.0) software, as described previously (Piccinini, 2015; Zanoni et al., 2016). AnaSP calculates the following: Volume, number of voxels of the volume of the foreground, reconstructed using the ReViSP (Piccinini et al., 2015) algorithm; Diameter, diameter of a circle with the same area as the foreground, computed as √(4×Area/π); Sphericity, as 4×π×Area/Perimeter, with ‘Area=area of the original mask’ and ‘Perimeter=perimeter of the original mask’; and Compactness, as 4×π×Area/Perimeter², with ‘Area=area of the original mask’ and ‘Perimeter=perimeter of the original mask’.

### Flow cytometry

Flow cytometry analysis was used to evaluate the expression of cell surface markers. Cells were incubated with the following monoclonal antibodies: anti-ESA (CD326)-PE (Miltenyi Biotec, MACS, clone HEA-125, cat. no. 130-113-826, 1:50), CD44-APC (Miltenyi Biotec, MACS, clone DB105, cat. no. 130-113-893, 1:50), CD24-FITC [eBioscience™, Invitrogen™, clone eBioSN3 (SN3 A5-2H10), cat. no. 15576356, 1:20], CD90-PE/Cy7 (BioLegend, clone 5E10, cat. no. 328123, 1:25), CD73-PE (BioLegend, clone AD2, cat. no. 344003, 1:25) and CD274-PE (BD Bioscience, clone MIH1, cat. no. 561787, 1:25). Cells were incubated for 20 min at 4°C with the recommended dilutions of each antibody, as per the manufacturer's guidelines. After staining, cells were washed twice with CellWASH (BD Bioscience, cat. no: 349524) and resuspended in CellWASH for analysis. Cells were collected with a BD LSR II flow cytometer (BD Bioscience) and analysed using BD FACS Diva (v6.2; BD Biosciences) and FlowJo (v10.9; BD Biosciences) software. Unstained cells were used as controls. For each sample, 20,000 events were collected.

### Drug toxicity assay

The ovarian cancer cell line strains were seeded in 96-well flat-bottom plates at a density of 9000 cells per well in 200 µl of supplemented drug-free medium. Cells were allowed to attach for 24 h at 37°C in a humidified atmosphere with 5% CO_2_.

The following day, the medium was replaced with fresh medium containing varying concentrations of cisplatin (0.03-100 µM), carboplatin (3-10,000 µM) and doxorubicin (0.003-10 µM), with each concentration tested in triplicate. Cells were incubated for 48 h under the same conditions. Cell viability was assessed using a Cell Counting Kit-8 (CCK-8; Dojindo, cat. no. NC9261855), following the manufacturer's protocol. Absorbance was measured at 450 nm using a BioTek ELx800 microplate reader (BioTek Instruments, Inc.). Control wells containing cell-free medium were included to account for background signal. The experiment was independently repeated two times. The IC50 and 95% CI values were obtained by fitting a four-parameter logistic model [log(inhibitor) versus response, variable slope] in GraphPad Prism (v8.0.1); between-strain *P*-values were derived with Prism's extra-sum-of-squares *F*-test.

### RNA extraction and quantification

Total RNA was extracted from the samples using TRIzol (Invitrogen™, cat. no. 15596018) and an RNeasy Mini Kit (Qiagen, cat. no. 74104), following the manufacturer's protocol. The concentration and purity of the extracted RNA were measured using a NanoDrop spectrophotometer (Thermo Fisher Scientific™ NanoDrop™ 2000). RNA sample absorbance was measured at 260 nm and 280 nm to calculate the RNA concentration. The 260/280 ratio was used as an indicator of RNA purity, with values between 1.8 and 2.0 considered acceptable. RNA samples were stored at −80°C until further use.

### Bulk RNA barcoding and sequencing, and data processing

Bulk RNA barcoding and sequencing, and pre-processing of the data (demultiplexing and alignment) was performed by Alithea Genomics, as described previously (Alpern et al., 2019). Bulk RNA-seq data were processed using R (v4.3.1). Gene expression counts were filtered to exclude lowly expressed genes using three counts per million in two or more samples as a threshold. Genes with the highest variance across samples (*N*=100) were selected to generate heatmaps for the evaluation of data quality and clustering. Log2-transformed counts per million (CPM) values were used for heatmap visualizations. Heatmaps were created using the ggplot2 package (Wickham, 2016).

### Differential gene expression and pathway enrichment analysis

Gene expression data normalization was performed using trimmed mean of M-values to correct for library composition biases (Robinson and Oshlack, 2010), followed by voom transformation. Linear models were fitted with lmFit, and, to account for variability and improve statistical power, eBayes from limma package was used (Ritchie et al., 2015). Differentially expressed genes (DEGs) were identified based on false discovery rate (FDR)-adjusted *P*-values (q-value<0.05) and log2 fold-change thresholds (|logFC|>1.5) for S1 versus S2 strains and 2D versus 3D cell cultures. Volcano plots of DEGs were generated using the EnhancedVolcano package. Genes with significant expression changes (FDR<0.05, |logFC|>1.5) are shown in darker red or blue colours in figures.

GSEA was performed using the clusterProfiler package. Gene sets were retrieved from the MSigDB database including hallmark pathways (H) *N*=50; Gene Ontology (GO) biological processes (C5: GO:BP) *N*=7608, cellular components (C5: GO:CC) *N*=1026 and molecular functions (C5: GO:MF) *N*=1820; Kyoto Encyclopedia of Genes and Genomes (KEGG) pathways (C2: KEGG) *N*=658; Reactome pathways (C2: CP:REACTOME) *N*=1736; WikiPathways (C2: CP:WIKIPATHWAYS) *N*=830; and oncogenic signatures (C6) *N*=189. DEGs were ranked based on log2 fold changes (logFC) derived from the differential expression analysis. For visualization of enrichment pathways, aPEAR package was used (Kerseviciute and Gordevicius, 2023). Dot plots illustrating the top 15 enriched pathways were created using ggplot2, with pathway size, network enrichment score and q-values mapped to point size and colour.

### Statistical analysis

Data normality was verified with the Shapiro–Wilk test and equality of variances with Levene's test. Unpaired, two-tailed Student’s *t*-test was used for pairwise comparison. Datasets with two independent factors were evaluated by two-way ANOVA followed by Tukey's multiple-comparison post-hoc test. Statistical analysis was performed using R (v4.3.1) or as specified in the previous paragraphs. *P*<0.05 was considered statistically significant.

## Supplementary Material

10.1242/dmm.052451_sup1 Supplementary information
