# Supplementary material for: Extended passaging of the SKOV3 ovarian cancer cell line leads to two phenotypically different strains
Source: Dis Model Mech. 2025 Aug 26;18(8):dmm052451. doi: 10.1242/dmm.052451 (PMC12421797; doi:10.1242/dmm.052451)
Supplement: Supplementary information [file dmm-18-052451-s1.pdf]

**Table S1.** The top 100 most variable genes between SKOV3 S1 and S2, full gene list.

| Gene_Symbol | SKOV3.S1_rep2 | SKOV3.S1_rep1 | SKOV3.S2_rep1 | SKOV3.S2_rep2 |
|---|---|---|---|---|
| SMR3B | 8.710854 | 8.801433 | 0.938371 | 0.978565 |
| MMP13 | 7.290692 | 7.500452 | -0.45021 | -0.12539 |
| OPRPN | 4.815397 | 4.602826 | -1.95363 | -1.95363 |
| AREG | 1.239432 | 2.355436 | 8.303361 | 8.247932 |
| SCEL | -0.89285 | -1.37317 | 5.122815 | 5.351467 |
| KRT81 | 6.263098 | 6.242327 | -0.23382 | 0.391672 |
| MRLN | -0.55906 | -1.37317 | 5.021239 | 5.130594 |
| FGFBP1 | 1.964648 | 1.940625 | 7.783488 | 7.810205 |
| EREG | -0.55906 | -0.63981 | 5.047311 | 4.889054 |
| MMP1 | 7.59577 | 7.691056 | 2.286121 | 2.203162 |
| LINC02904 | -1.95363 | -1.95363 | 3.400904 | 3.378579 |
| AMTN | 11.25161 | 11.13816 | 5.832693 | 5.90195 |
| EDIL3 | 7.120903 | 7.210271 | 1.738844 | 2.161293 |
| AP1M2 | -0.55906 | -1.95363 | 3.819256 | 3.761469 |
| TC2N | -0.55906 | -1.37317 | 4.103957 | 4.151917 |
| TMTC1 | 3.891245 | 4.044266 | -1.40886 | -0.61414 |
| DIO2 | 4.530419 | 4.587561 | -0.23382 | -0.61414 |
| COL8A1 | 6.047642 | 5.890867 | 0.642503 | 1.394567 |
| NREP | 5.865373 | 5.90325 | 0.748013 | 1.071916 |
| DPP4 | -1.32786 | -1.37317 | 3.448638 | 3.598463 |
| TM4SF18 | 3.228155 | 2.915932 | -1.40886 | -1.95363 |
| SLC22A2 | 3.184329 | 3.010539 | -1.95363 | -1.35888 |
| P2RY6 | 4.343814 | 4.12049 | -0.0457 | -0.93898 |
| IGFBP5 | 7.754049 | 7.706153 | 2.734237 | 3.264563 |
| COL5A1 | 7.754986 | 7.669469 | 3.013531 | 2.981457 |
| FOXS1 | 3.27069 | 3.281113 | -1.95363 | -0.61414 |
| EPHA4 | 3.748062 | 3.390711 | -0.70488 | -1.35888 |
| DACT2 | -1.95363 | -0.96039 | 3.095782 | 3.097008 |
| GALNT14 | -1.32786 | -1.37317 | 3.034537 | 3.396735 |
| LRATD1 | 3.502507 | 3.408195 | -1.40886 | -0.61414 |
| IGFL2-AS1 | 6.538003 | 6.546592 | 1.738844 | 2.396013 |
| PF4V1 | 2.652246 | 2.391043 | -1.95363 | -1.95363 |
| SUCNR1 | 2.517447 | 3.010539 | -1.40886 | -1.95363 |
| NCALD | -0.89285 | -1.37317 | 3.432901 | 3.183217 |
| FGF5 | 3.115987 | 3.373013 | -1.40886 | -0.93898 |
| MUC5AC | -1.32786 | -0.96039 | 3.282971 | 3.203987 |
| LAMA3 | 4.094345 | 3.963788 | 8.476936 | 8.32758 |
| CNRIP1 | 4.573528 | 4.290393 | -0.23382 | 0.529702 |
| BMF | 5.288785 | 5.242373 | 0.748013 | 1.242243 |
| PCDH7 | 3.622314 | 3.801261 | -0.45021 | -0.61414 |
| RAB34 | 1.546871 | 2.318927 | 6.100128 | 6.173456 |
| CLDN11 | 1.15112 | 1.034527 | 5.231418 | 5.419647 |
| MDFI | -0.89285 | -0.37768 | 3.757423 | 3.378579 |

Disease Models & Mechanisms • Supplementary information

| | | | | |
|---|---|---|---|---|
| SLC16A2 | 3.249579 | 3.242648 | -1.01435 | -0.93898 |
| ESRP2 | 0.464362 | -0.63981 | 4.162382 | 3.856364 |
| KIT | 2.481683 | 2.459725 | -1.40886 | -1.95363 |
| DAB2 | 4.77164 | 4.827366 | 0.748013 | 0.529702 |
| TGFB2 | 3.206409 | 2.940171 | -1.40886 | -0.61414 |
| H1-0 | 2.202931 | 2.459725 | 6.49814 | 6.39962 |
| LSAMP | -0.89285 | -1.95363 | 2.857242 | 2.35945 |
| AKT3 | 4.293132 | 4.212924 | -0.0457 | 0.391672 |
| MOB3B | 0.731263 | -1.37317 | 3.384635 | 3.582689 |
| PSG1 | 4.476927 | 4.192889 | 0.748013 | -0.12539 |
| CCL20 | 3.161906 | 3.299967 | 7.302917 | 7.262832 |
| VEGFC | 3.670763 | 4.212924 | -0.45021 | 0.391672 |
| COL5A2 | 6.225751 | 6.379383 | 1.974489 | 2.659288 |
| PIK3CG | 1.911877 | 2.162888 | -1.95363 | -1.95363 |
| CDH6 | 6.324955 | 6.187693 | 2.286121 | 2.243849 |
| NOS1 | -1.32786 | -1.95363 | 2.542251 | 2.027862 |
| DEFB1 | -1.32786 | -1.95363 | 2.354541 | 2.243849 |
| TNFSF10 | 7.281621 | 7.338911 | 3.351537 | 3.432377 |
| F2R | 6.941655 | 6.893944 | 3.075655 | 2.932514 |
| NPFFR2 | 0.136613 | 0.941531 | 4.358387 | 4.458888 |
| FA2H | 0.603976 | 0.620055 | 4.574602 | 4.441501 |
| UNC13D | 1.679069 | 2.243016 | 5.879885 | 5.747813 |
| PSG9 | 2.889083 | 2.866195 | -1.01435 | -0.93898 |
| DLGAP1 | -1.95363 | -1.37317 | 2.320737 | 1.980494 |
| ANPEP | 5.544175 | 5.467534 | 1.837766 | 1.532334 |
| CRABP1 | 0.464362 | 1.204263 | 4.623947 | 4.614377 |
| UGT8 | -1.95363 | -0.63981 | 2.176994 | 2.597835 |
| LINC01638 | 4.106137 | 3.998829 | 0.269879 | 0.239024 |
| EFEMP1 | 7.909343 | 7.923866 | 4.000996 | 4.25504 |
| RAB39B | -1.95363 | -0.63981 | 2.387572 | 2.321936 |
| MT1B | 3.821429 | 3.903425 | -0.45021 | 0.878754 |
| CLDN16 | 1.057048 | 0.49473 | 4.470541 | 4.550938 |
| PDE5A | 1.857101 | 2.525285 | -1.95363 | -0.93898 |
| ALPK2 | 5.009979 | 4.669589 | 1.576448 | 0.77152 |
| OLFML3 | 3.27069 | 3.281113 | -0.70488 | -0.12539 |
| SPARC | 8.8043 | 8.943477 | 4.842195 | 5.555148 |
| CCDC80 | 7.795606 | 7.761614 | 4.011629 | 4.130377 |
| RHOBTB1 | 3.332229 | 3.476084 | -0.45021 | -0.12539 |
| SERPINB5 | 3.312006 | 3.20313 | 0.269879 | -0.93898 |
| GPRIN2 | -0.0602 | -0.37768 | 3.384635 | 3.501122 |
| KAZN-AS1 | 2.112284 | 2.12109 | -1.01435 | -1.95363 |
| GASK1B | 3.589087 | 3.425469 | -0.45021 | 0.239024 |
| FNDC1 | 3.589087 | 3.442538 | 0.120702 | -0.34916 |
| ANGPTL4 | 6.231146 | 6.332079 | 2.542251 | 2.746822 |
| RASAL1 | -1.95363 | -0.15594 | 2.451447 | 2.243849 |
| LRRC38 | 1.964648 | 2.391043 | -0.70488 | -1.95363 |
| DMKN | 2.833418 | 2.425793 | 6.266956 | 6.204709 |
| CD70 | 9.420465 | 9.470324 | 5.868231 | 5.793068 |
| ICAM2 | -0.28818 | 0.735358 | 3.705975 | 3.802905 |
| LRRN1 | 3.589087 | 3.540921 | -0.23382 | 0.239024 |
| LINC01711 | 2.202931 | 2.203508 | -0.45021 | -1.95363 |

Disease Models & Mechanisms • Supplementary information
